# Arthro-Broström with endoscopic retinaculum augmentation using all-inside lasso-loop stitch techniques

**DOI:** 10.1186/s12891-022-05709-8

**Published:** 2022-08-20

**Authors:** Yunjian Yang, Jing Han, Helin Wu, Xiaosong Zhi, Junhong Lian, Feng Xu, Xianhua Cai, Shijun Wei

**Affiliations:** 1 Department of Orthopaedics, General Hospital of Central Theater Command, NO. 627, Wuluo Road, Wuhan, 430030 Hubei Province People’s Republic of China; 2 The First Clinical Medical School Of, Southern Medical University, Guangzhou, Guangdong Province People’s Republic of China

**Keywords:** Arthroscopy, Endoscopy, Anterior talofibular ligament, Chronic ankle instability, Inferior extensor retinaculum, Lasso-loop stitch

## Abstract

**Background:**

There is still some controversy about the augmentation of the inferior extensor retinaculum after arthroscopic anterior talofibular ligament repair. The aim of this study was to evaluate the novel arthro-Broström procedure with endoscopic retinaculum augmentation using all-inside lasso-loop stitch techniques for chronic lateral ankle instability.

**Methods:**

Thirty-four cases with grade-2 or grade-3 chronic anterior talofibular ligament lesions who underwent the novel arthro-Broström procedure with endoscopic retinaculum augmentation using all-inside lasso-loop stitch techniques were assessed retrospectively. A total of 30 cases (30 ankles) were followed up for a mean of 26.67 ± 4.19 months (range, 24—36 months). four cases were excluded due to insufficient medical records or loss of follow-up reports. The Cumberland Ankle Instability Tool scores, The Karlsson-Peterson scores and Visual Analogue Scale scores were evaluated before surgery and at the final follow-up time. Also, the results of stress fluoroscopic tests and complications were recorded.

**Results:**

At the final follow-up, the average of the Cumberland Ankle Instability Tool scores, The Karlsson-Peterson scores and Visual Analogue Scale scores were 86.63 ± 6.69 (range, 77—100), 90.17 ± 4.64 (range, 85—100) and 0.53 ± 0.63 (range, 0—2), respectively. Moreover, the results of stress fluoroscopic tests were improved significantly after surgery. Mild keloid formation and/or knot irritation were observed in four cases. No wound infections, nerve injuries and recurrent instability were recorded. Also, no stiffness or arthritis of the subtalar joint was encountered.

**Conclusions:**

The arthro-Broström procedure combined with endoscopic retinaculum augmentation using all-inside lasso-loop techniques is reliable and safe due to the advantage of direct endoscopic visualization.

## Background

Lateral ankle ligament injuries are by far the most common injuries due to people’s active lifestyles, however, around 20% of cases should be treated with operative procedures after conservative methods have not been successful [1, 2]. The goal of this surgical treatment is restoration of ankle stability and function while minimizing complications. The modified Broström-Gould procedure has been considered the gold standard for chronic lateral ankle instability (CLAI) over the last five decades [1–3]. These procedures were gradually developed from formal open techniques to minimally invasive ones over the years. However, there are still debates related to the necessity and reliability concerning the augmentation of the inferior extensor retinaculum (IER) after arthroscopic anterior talofibular ligament (ATFL) repair [4–6]. For those cases with an insufficient ATFL remnant, however, the augmentation of the IER is an additional choice, or ligament reconstruction [7–9]. Guillo et al. first reported the technique note of all-inside endoscopic Broström-Gould procedure without clinical results [10]. Then, Cordier et al. described that the all-arthroscopic Broström-Gould technique using an automatic suture passer had shown excellent clinical results with short-term follow-ups [11]. Actually, in most percutaneous or arthroscopic assisted Broström-Gould procedures, these sutures were directly passed through the IER and then stitched into a horizontal mattress configuration, the reliability of this basic reinforcement method is most vulnerable, due to the location and quality of the IER in each individual case [1, 12–15].

The aim of this study is to evaluate a novel arthro-Broström procedure combined with endoscopic retinaculum augmentation using all-inside lasso-loop stitch techniques for chronic lateral ankle instability. We have hypothesized that restoration of the ankle stability and function can be achieved by this all-inside lasso-loop stitch technique while avoiding the risk of nerve injuries.

## Methods

### Patients

The present study is a retrospective investigation of 30 consecutive active patients with chronic lateral ankle instability, who underwent the arthro-Broström procedure with endoscopic retinaculum augmentation. Inclusion criteria [9, 16]: (1) recurrent ankle sprain and signs of ankle instability; a positive or questionable result is associated with anterior drawer and talar tilt stress fluoroscopy (Ligs device, innomotion inc. Shanghai, China); (2) no response to conservative treatments for over three months; (3) the grade-2 or grade-3 lesion of the anterior talofibular ligament (ATFL) was indicated by magnetic resonance imaging (MRI) and ultrasonography (US). Exclusion criteria: (1) a combined ligament injury with an acute fibular fracture, previous ankle surgery, any foot and ankle malalignment; (2) The absence, severely scared or calcified ATFL remnant was confirmed under arthroscopic examination (grade-4 lesion). (3) The presence of generalized ligamentous laxity. (4) Patients with systemic diseases, neuromuscular disorders and infectious diseases. The study was approved by the institutional ethical committee of our hospital. All procedures performed in studies involving human participants were in accordance with the ethical standards of the institutional and/or national research committee and with the 1964 Helsinki Declaration and its later amendments or comparable ethical standards. An informed consent was taken from all patients before their participation in this study.

Thirty-four cases were treated with this technique in our institution from August 2018 to October 2019. Four cases were excluded due to insufficient medical records or loss of follow-up data. A total of 30 cases (30 ankles) were followed up for a mean of 26.67 ± 4.19 months (range, 24–36 months). Of these cases, 5 were female (16.67%), 25 were male (83.33%), 14 right ankles (46.67%) and 16 left ankles (53.33%). The average age was 31.83 ± 6.90 years (range, 21–46 years), with 8 being smokers. Concomitant intra-articular pathologic findings including: bone impingement (*n* = 9, 30%), osteochondral lesion (OCL) (*n* = 8, 26.67%), osseous avulsion of the fibular (*n* = 4, 13.33%), os subfibulare (*n* = 1, 3.33%), and loose body (*n* = 2, 6.67%). According to the classification of the ATFL lesion by Thès [9], there were 11 grade-2 cases (36.67%) and 19 grade-3 cases (66.33%). The average distance between the proximal margin of the IER and anterior margin of the lateral malleolus is 8.8 ± 2.58 mm (range, 5–15 mm). According to the medical records, the average operative time was 59.07 ± 9.81 min (range, 43–74 min). Those cases with concomitant pathology such as bone impingement, OCL and osseous avulsion of the fibular needed more time. Demographic data is shown in Table 1.

**Table 1:** Patients’ characteristics

Variable	Total, *n* = 30
Age	31.83 ± 6.90
Gender	
Male	25 (83.33)
Female	5(16.67)
Occupation	
Soldier	16(53.33)
Athlete	4(13.33)
Other	10(33.33)
Injury side	
Left	16(53.33)
Right	14(46.67)
Concomitant intra-articular pathology	
Bone impingement	9(30)
Osteochondral lesion	8(26.67)
Osseous avulsion of the fibula	4(13.33)
Os subfibulare	1(3.33)
Loose body	2(6.67)
Distance between IER and LM (mm)	8.8 ± 2.58
The operative time(min)	59.07 ± 9.81
Follow up time (mth)	26.67 ± 4.19

Categorical variables were reported as number (n) and percentage (%)

Continuous variables are reported as mean ± SD

*LM* Lateral malleolus, *IER* Inferior extensor retinaculum

### Surgical procedures

**Fig. 1:**
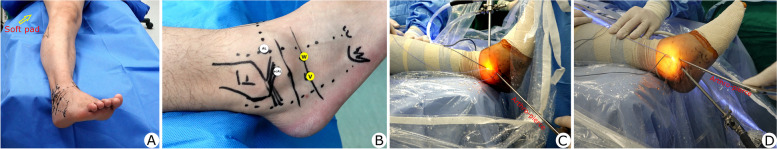
**a** The patient was placed in the supine position, the ipsilateral back and hip was elevated with a soft pad. **b** The viewing portal for the IER was located at the mid-distance point between the tip of the lateral malleolus and the fifth metatarsal base. An accessory working portal was created 1.5 cm superior to this portal. **c** The ankle was positioned in a neutral position for the ATFL repair. **d** The ankle was then placed in a slight medial rotation position for augmentation of the IER

Set up the patient in the supine position, the ipsilateral hip were elevated with a big soft pad to place the ankle in a slight medial rotation (Fig. 1A). A tourniquet was placed at the proximal thigh, a traction device was unnecessary. The anatomic landmarks were outlined along the medial and lateral malleoli, the tibialis anterior tendon, peroneus tertius tendon and peroneal tendon. Also, the general orientation line of the inferior extensor retinaculum (IER) was palpated and drawn which is located at roughly 1–1.5 cm anterior to the tip of the lateral malleolus (Fig. 1B). The lower extremity was prepped in the usual sterile fashion with a complex iodine solution.

### Step-one: All-inside arthroscopic Broström procedures

**Fig. 2:**
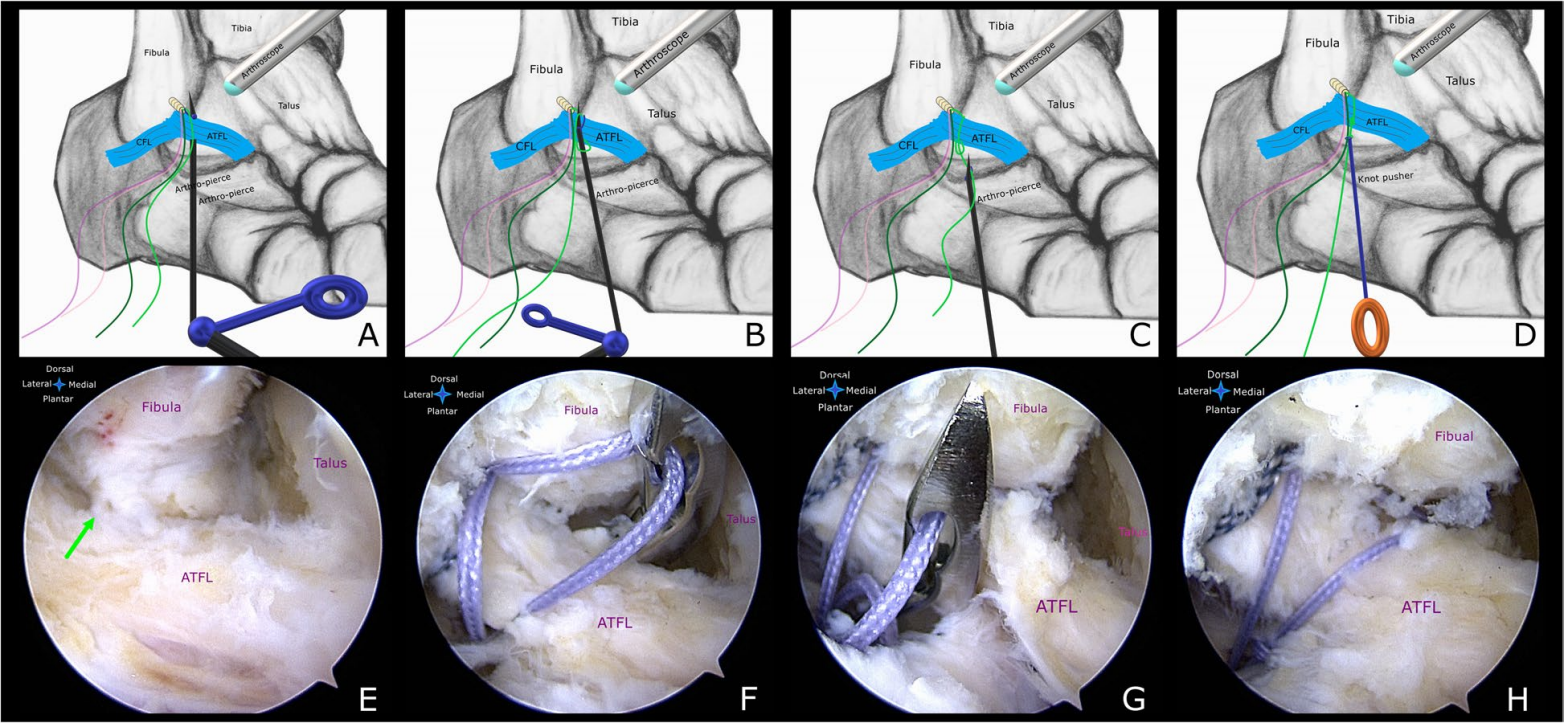
**a,e,f** The Arthro-Pierce instrument was introduced into the center of the triangle space bordered by the lateral malleolus, the lateral talar articular surface and the ATFL via the acAL portal. One limb of the violet strands (*shown in green in Fig. 2A,B,C,D*) has been grasped. The Arthro-Pierce instrument was slowly retrieved. This limb followed through the ATFL, then a loop was created. **b,c,g** The Arthro-Pierce instrument was passed through the created loop. The free end of this limb was then grasped. **d** and **h** A self-cinching stitch of the ATFL was created. The ligament remnant was then draw toward the bone surface when the axial thread (*shown in dark green in Fig. 2A,B,C,D*) was pulled back

The all-inside lasso-loop stitch technique was performed for the torn ATFL talar remnant, which was described previously [17]. Firstly, the ankle was placed in neutral position by the assistant, then the modified anteromedial (AM) portal was established (Fig. 1C). The 4.0 mm arthroscopic cannula and trocar was inserted while the tibialis anterior tendon was pushed laterally, so, visualization of the lateral gutter could be enhanced. Next, the anterolateral (AL) portal was typically created using an outside-in technique. The associated intra-articular lesions were treated at this stage. The lateral gutter was then debrided gently using a small size end-cutting shaver, afterward, the ATFL remnant was evaluated for quality with a probe. Under arthroscopic visualization, an accessory anterolateral (acAL) portal which is approximately 1.0 cm anterior to the tip of the lateral malleolus was then created. The fibular insertion site of the ATFL was gently prepared with a small burr. A 3.0 mm absorbable suture anchor (GRYPHON™ BR Anchor, Johnson & Johnson, USA), which was preloaded with two different color strands of #2 sutures, was implanted into the fibular footprint of the ATFL using a drill guide via the acAL portal. The striped, colored strands were retrieved from the AL portal, these will be used to reinforce the IER later. Under arthroscopic visualization, the maximum possible amount of the ATFL remnant was penetrated by the Arthro-Pierce instrument (Curved, Smith & Nephew, USA) via the acAL portal. Advance the tip of this instrument until it is in the center of the triangle space bordered by the lateral malleolus, the lateral talar articular surface and the ATFL (Fig. 2A,E). When a limb of the violet strands has been grasped, the Arthro-Pierce instrument was slowly retrieved. This limb was pulled through the ATFL remnant while a loop of this limb was created in the lateral gutter (Fig. 2B,F). The Arthro-Pierce instrument was then rotated in the opposite direction to release the created loop. The tip of this instrument was passed through this loop, the free end of this limb was then grasped and passed through it (Fig. 2C,G). A self-cinching stitch of the ATFL was created when this limb was pulled tight. Finally, the stitched ATFL remnant was then pulled toward the bone surface when the other free end of the violet strands was retrieved. The surgical knot was created and tightened 2–3 times using a knot pusher while holding the ankle in a neutral position (Fig. 2D,H).

### Step-two: All-inside endoscopic retinaculum augmentation procedure

**Fig. 3:**
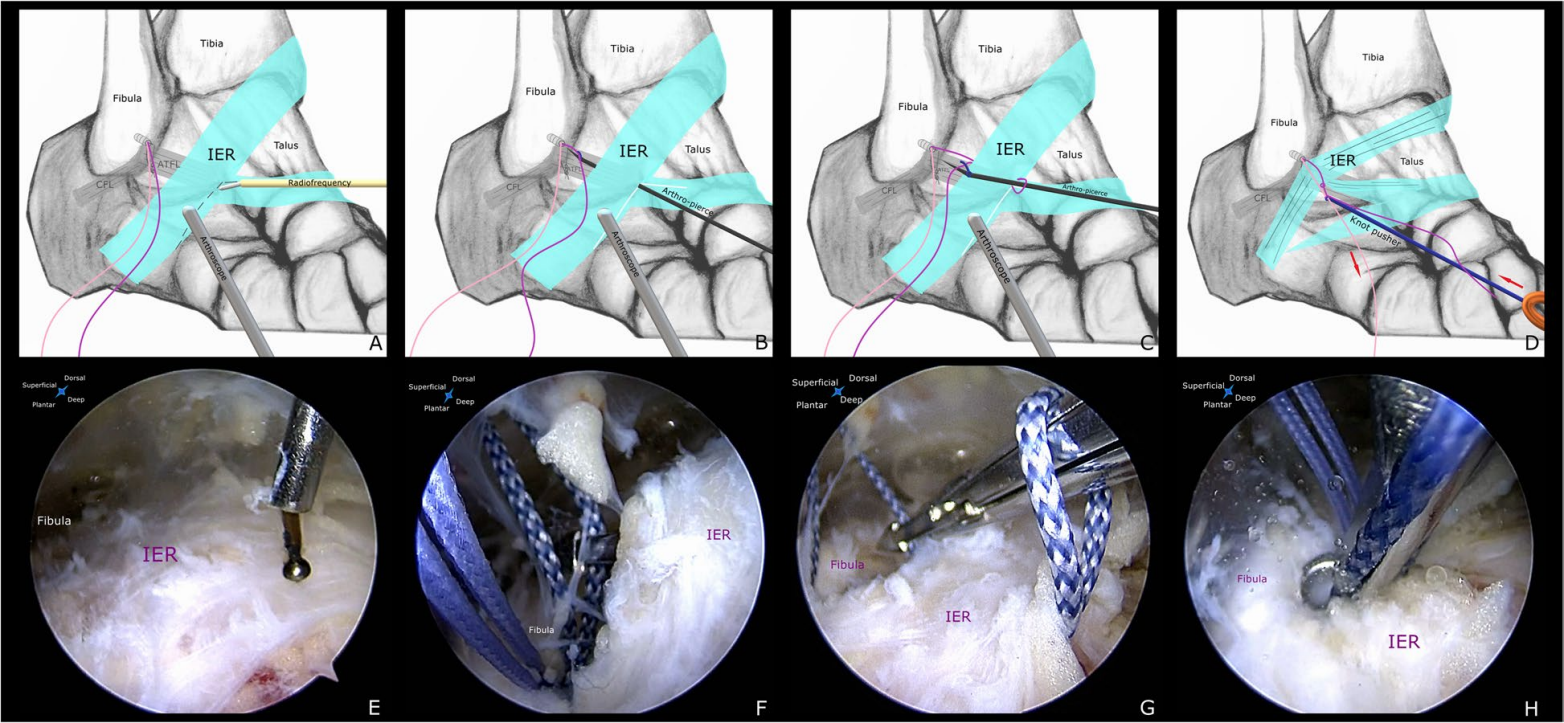
**a** and** e** The IER was released along the distal edge of the stem using a small-size radiofrequency device. **b** and **f** The Arthro-Pierce instrument was passed through the deep layer of the IER. The tip of this instrument was advanced until one limb of the striped, colored strands (*shown in violet in Fig. 3A,B,C,D*) was grasped. The Arthro-Pierce instrument was then slowly retrieved. **c** and **g** When a loop of this limb was created, this instrument was then released and passed through this loop to grasp the free end. A self-cinching stitch of the retinaculum was created. **d** and **h** The stitched retinaculum was then drawn toward the lateral malleolus when this axial strand(*shown in pink in Fig. 3A,B,C,D*) was pulled back

**Fig. 4:**
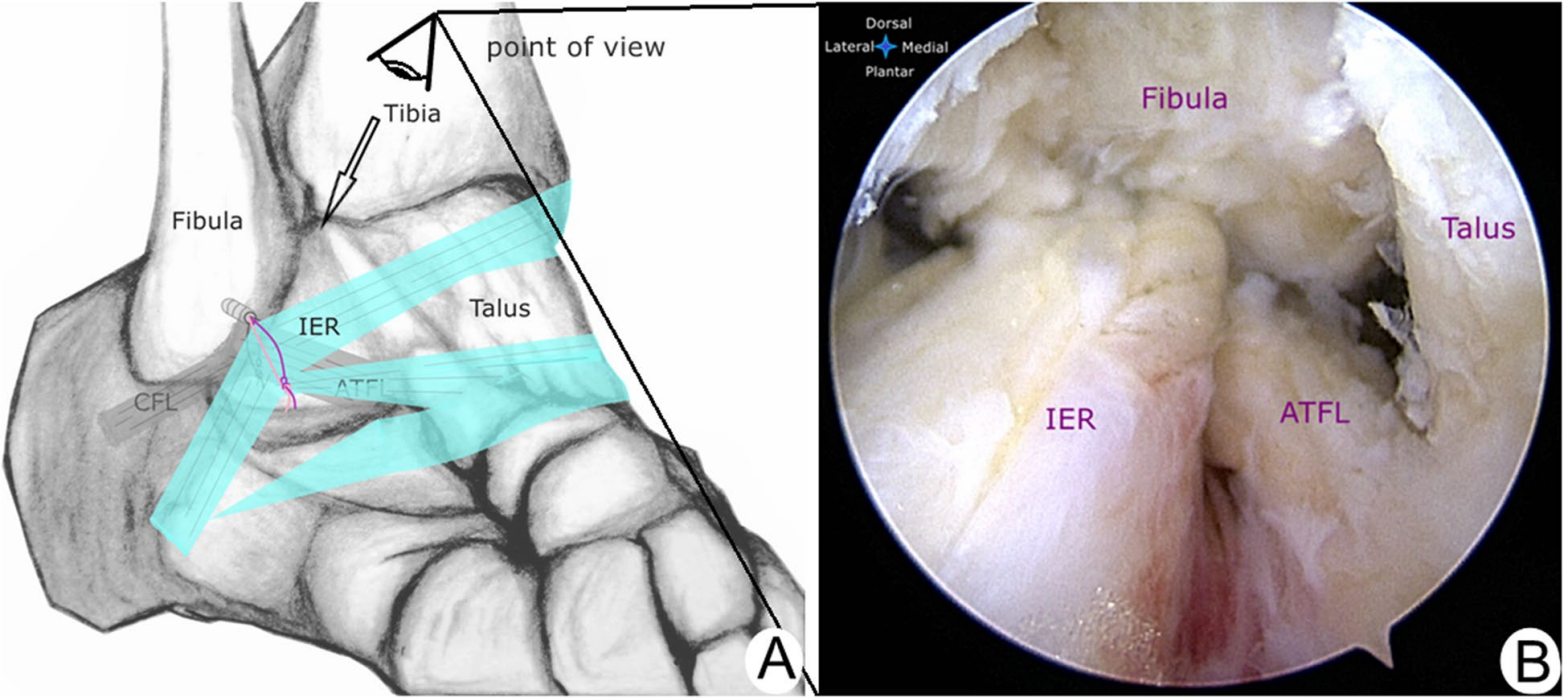
**a** and **b** Under arthroscopic visualization, the appropriate tension of the augmented IER was confirmed

The ankle was then placed back to a slight medial rotation position (Fig. 1D). The viewing portal for the inferior extensor retinaculum (IER) was created at the mid-point between the tip of the lateral malleolus and the fifth metatarsal base [13]. A working space was created between the superficial fascia and the retinaculum using a small mosquito. Another accessory portal for working instruments was created 1.5 cm superior to the viewing portal using an outside-in technique (Fig. 1B). The margins of the lateral malleolus and the IER were exposed gently using the 2.9 mm end-cutting shaver. The striped, colored strands and the ATFL repaired site were cleared carefully. The IER was released along the distal edge of the stem using a small-size radiofrequency device (Fig. 3A,E). So, it can be brought to the anterior margin of the lateral malleolus with the desired tension. The Arthro-Pierce instrument was then passed through the deep layer of the retinaculum under endoscopic visualization. The tip of this instrument was advanced until one limb of striped, colored strands was grasped (Fig. 3B,F). The Arthro-Pierce instrument was then slowly retrieved. This limb was pulled through the deep layer of the retinaculum. When a loop of this limb was created, this instrument was then released and passed through this loop to grasp the free end of this limb (Fig. 3C,G). A self-cinching stitch of the retinaculum was created when the free end of this limb was pulled tight. Also, the other free end of the striped, colored strand (the axial strand) was grasped and pulled to the accessory working portal. The stitched retinaculum was then drawn toward the lateral malleolus when this axial strand was pulled back (Fig. 3D,H). Under endoscopic visualization, this suture was tied using a surgical knot with the ankle in a neutral position. The IER was then attached to the lateral malleolus with appropriate tension (Fig. 4A,B). These strands were cut and the skin incisions were closed.

### Postoperative management

Postoperative management consists of protecting the repaired site with a semi-rigid ankle brace. Full weight bearing and active range of motion exercises are allowed with this brace at one week postoperatively. Inversion of the ankle should be prohibited before four weeks postoperatively. At three months, most casual sport activities are allowed with elastic tape support. Competitive sport activities could be performed after six months depending on patients’ evaluations.

### Clinical outcome evaluation

Patients were scheduled for evaluation at postoperative 3, 6, 12, 18, 24 months and beyond. Anterior drawer tests and talar tilt tests were examined by stress fluoroscopy before surgery and at the final follow-up. The clinical outcomes were assessed using the Cumberland Ankle Instability Tool (CAIT), Karlsson-Peterson score and Visual Analogue Scale (VAS) score at the preoperative evaluation and the final follow-up [18–20]. The clinical outcomes and complications were recorded by an independent surgeon with a physical therapist.

### Statistical analysis

Statistical analysis was performed using SPSS 16.0 (SPSS Inc, Chicago, IL). Wilcoxon signed-rank test was conducted for evaluation of changes in the Cumberland Ankle Instability Tool (CAIT) scores, Karlsson-Peterson scores and VAS scores before surgery and at the final follow-up. P values less than 0.05 were considered to be statistically significant.

## Results

### Functional outcomes

**Fig. 5:**
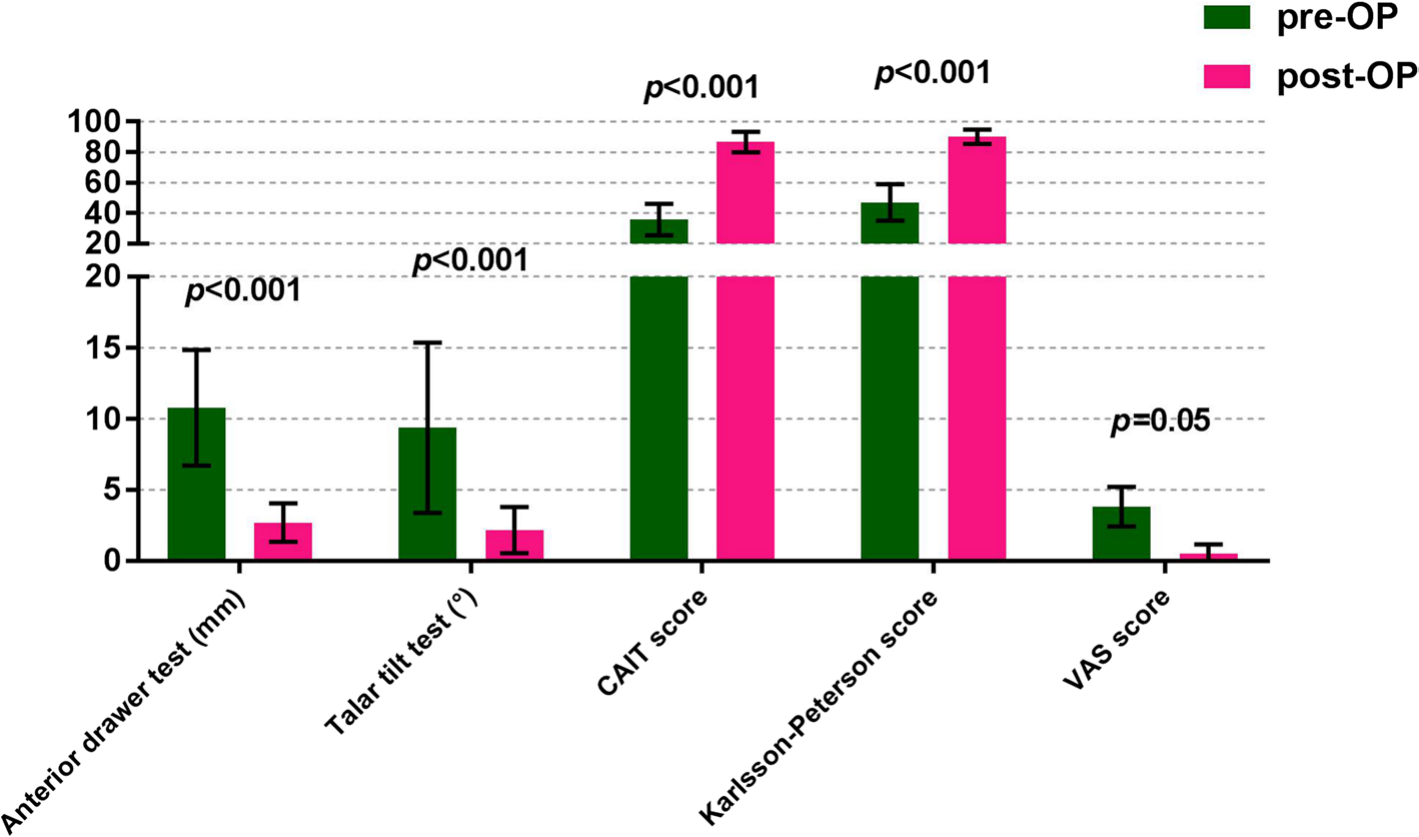
The Cumberland Ankle Instability Tool (CAIT) scores and the Karlsson-Peterson scores were improved significantly at the final follow-up (*P* < 0.001). The average VAS score was decreased at the final follow-up (*P* = 0.05). Moreover, the results of stress fluoroscopic tests were improved significantly after surgery (*P* < 0.001)

**Fig. 6:**
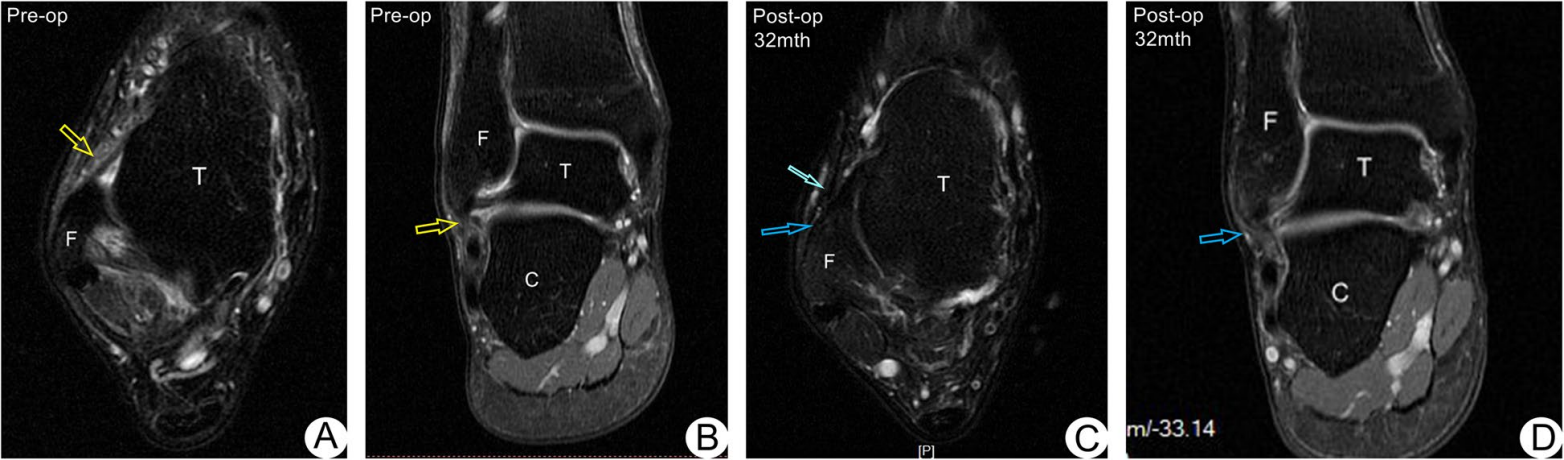
Female, 33 years old, had a sprained left ankle one year ago. **a** and **b** The axial and coronal T2-weighted images show the chronic lesion of the ATFL and CFL (*yellow arrows*). **c** and **d** The axial and coronal T2-weighted images show the appropriate regeneration of the ATFL and CFL (*bule arrows*) at 32 months postoperative. The reinforced IER was still attached at the lateral malleolus (*light bule arrows*). No degeneration of the subtalar joint was encountered

The Cumberland Ankle Instability Tool (CAIT) scores improved significantly from an average preoperative score of 35.77 ± 10.32 points (range, 13–47) to 86.63 ± 6.69 points (range, 77—100) (*P* < 0.001). Also, the Karlsson-Peterson scores increased significantly from an average preoperative score of 47.1 ± 11.99 points (range, 27—65) to 90.17 ± 4.64 points (range, 85—100) at the final follow-up (*P* < 0.001). The average VAS score decreased from 3.73 ± 1.55 points (range, 0—7) preoperatively to 0.53 ± 0.63 points (range, 0—2) postoperatively at the final follow-up (*P* = 0.05). The stress fluoroscopy performed at the final follow-up showed anterior talar translation decreased from 10.77 ± 4.07 mm (range, 4—19) preoperatively to 2.7 ± 1.34 mm (range, 0—5) postoperatively, and a talar tilt angle of 2.17° ± 1.62° (range, 0°—4°) against a 9.37° ± 5.98° (range, 3°—30°) angle preoperatively (*P* < 0.001). According to the patients’ survey, all patients returned to their previous level of sport activities at six months postoperatively. No recurrent ankle instability was encountered (Table 2, Fig. 5). Compared with the pre-surgery images (Fig. 6A, B), the outlines of the ATFL and CFL were defined on the MRI images at the final follow-up (Fig. 6C, D).

**Table 2:** Karlsson-Peterson scores, Cumberland Ankle Instability Tool (CAIT) scores, VAS scores, Stress fluoroscopy, Complications in Patients(*n* = 30)

Variable	Pre-op	Post-op	95% CI	p
Karlsson-Peterson score	47.10 ± 11.99	90.17 ± 4.64	-46.3 to -39.8	< 0.001
Cumberland Ankle Instability Tool (CAIT) score	35.77 ± 10.32	86.63 ± 6.69	-54.1 to -47.6	< 0.001
VAS score	3.73 ± 1.55	0.53 ± 0.63	0.04 to 6.56	0.05
Anterior drawer stress fluoroscopy(mm)	10.77 ± 4.07	2.70 ± 1.34	4.81 to 11.3	< 0.001
Talar tilt stress fluoroscopy (゜)	9.37 ± 5.98	2.17 ± 1.62	3.94 to 10.5	< 0.001
**Complications (n)**				
Infection	0 (0.0)			
Nerve injury	0 (0.0)			
Recurrent instability	0 (0.0)			
Stiffness or degeneration of the subtalar joint	0 (0.0)			
Anchor or Knot issue	2 (0.07)			
Keloid formation	3 (0.10)			

Categorical variables are reported as number (n) and percentage (%)

Continuous variables are reported as mean ± SD

*CI* Confidence intervals

VAS, Visual Analogue Scale

### Complications

One female case with poor bone quality was changed to a mini-incision repair with a 4.5 mm PEEK suture anchor, due to the previous anchor being pulled out when the IER was tied during surgery. No nerve injuries or wound infections were encountered. Three cases complained about discomfort around the released site of the IER with mild limitation of inversion of the ankle within three months postoperatively; the mild keloid formation was palpated around this area. Knot irritation was encountered in a female patient with a low body mass index (BMI). Those local symptoms were significantly relieved after six months without a second intervention. At the final follow-up, no stiffness or arthritis of the subtalar joint was observed (Table 2, Fig. 6D).

## Discussion

The important finding of this study is that the arthro-Broström procedure combined with endoscopic retinaculum augmentation using all-inside lasso-loop techniques is reliable and safe. The goal of the ligament reattachment should be focused on the durable biomechanical stability and improving ligament-bone interfaced healing. Even though the modified Broström-Gould repair technique was considered the gold standard, there is still some controversy on the augmentation of the inferior extensor retinaculum (IER) [5, 6, 21, 22]. Behrens reported that there is no significant biomechanical difference between traditional Broström repair and the modified Broström-Gould repair; reinforcement of the IER may be a marginal procedure at the time of surgery [23]. However, according to another classic biomechanics research, the augmentation of the IER provided protection to the primary ATFL repair within ankle inversion conditions [24]. Moreover, elongation of the repaired ATFL was significantly higher with unprotected motion of the ankle [25]. In a long-term clinical study of arthroscopic-assisted Broström-Gould repair, arthroscopic ATFL repair with IER augmentation can provide similar results as the gold standard ATFL and CFL repair. At the last appointments, 86.7% of active patients practiced normal sports activities at the same preinjury level without major complications [14]. Cordier et al. described the all-arthroscopic Broström-Gould technique using an automatic suture passer, excellent clinical results were observed with a median follow-up time of 28 months while only one case required a revision surgery [11]. According to previous studies, grade-1 and most grade-2 ATFL lesions only reattachment surgery was required without IER augmentation [9, 17, 26]. Actually, for grade-3 ATFL lesions and some high-level athletes with grade-2 ATFL lesions could be reinforced with IER. For those patients with grade-4 ATFL lesions or generalized ligamentous laxity, the ligament reconstruction, in my opinion, is the good choice. The reason why we don’t suggest ligament reattachment is because there is a higher risk of rerupture due to the poor quality of the ligament remnant [2, 3].

Actually, in most series with percutaneous or arthroscopic Broström-Gould techniques, once passed through the IER, the sutures are then tied into a horizontal mattress configuration [1, 13–15]. This basic stitch method may have influenced the reliability and durability of the reattached retinaculum. Furthermore, these procedures are most vulnerable, depending on the location and quality of the IER [12]. According to Takao et al.’s recent study, weight bearing was safe, after day one, using the modified lasso-loop technique for arthroscopic ATFL repair [26]. In our series, all-inside lasso-loop stitch techniques were used to improve tissue reattachment of the ATFL remnant and the IER. At the final follow-up, the mean Cumberland Ankle Instability Tool (CAIT) scores, The Karlsson-Peterson scores were 86.63 ± 6.69 and 90.17 ± 4.64, respectively. Moreover, the results of stress fluoroscopic tests were improved significantly after surgery. According to the patients’ survey, all patients returned to their previous level of sport activities. Fortunately, no recurrent ankle instability was encountered at the final follow-up.

Additionally, the stem of the IER could be accurately identified and separated under endoscopic visualization in the present study. According to Jeong’s previous report, the IER reinforcement wasn’t feasible in some cases (*n* = 10, 24.4%) due to the distance being too far between the tip of the fibular and the IER, moreover, the IER wasn’t found in 6 cases. In his study, the average distance between the IER and the tip of the fibular was 9.8 mm (range, 5–22 mm); when this distance was longer than 18 mm, reinforcement of the IER was not possible [21]. Actually, the structure used to reinforce the ATFL repair in most reported cases is the sural fascia, not the stem of the IER. The sural fascia is probably easy to tear during early rehabilitation. Dalmau-Pastor et al. reported that the stem of the IER, the only part that could be used in the augmentation of an ATFL repair, is often difficult to identify and reattach through a limited small incision or percutaneous procedure [27]. In our cases, the average distance between the proximal margin of the IER and anterior margin of the lateral malleolus is 8.8 ± 2.58 mm (range, 5–15 mm). This distance was longer than 10 mm only in 6 cases. Performing the sufficient augmentation of the IER was obtained due to accurate separation of the IER under endoscopic visualization, even though two cases with a thin stem of the IER were observed during surgery. In addition, the released IER was attached to the anterior margin of the lateral malleolus (near the footprint of the ATFL) not the tip of the fibular. Appropriate tension of the augmentation, not only protects the repaired ATFL remnant, but also avoids the avulsion of the reinforced IER during postoperative rehabilitation (Fig. 6C).

When arthroscopic assisted repaired techniques were applied, the entrapment of the superficial peroneal nerve or sural nerve is another important issue. The concept of a “safe zone” includes the distal fibula, the superior margin of the peroneal tendons and the intermediate branch of the superficial peroneal nerve was recommended to prevent this problem [1]. In a recent study, however, some small collateral branches of the cutaneous nerves were found crossing into the “safe zone” [28]. The close relationship of the IER with surrounding nerves is still a critical point to consider when performing a modified Broström-Gould procedure [29]. According to Guelfi et al.’s study, compared with arthroscopic all-inside repair techniques, a higher rate of neuritis was encountered in the arthroscopic-assisted technique [30]. So, the key point for avoiding nerve injuries is performing all procedures under direct visualization. In the present study, the absence of nerve injuries could also be explained by the all-inside procedures.

Finally, tightness of the subtalar joint after reinforcement of the IER to the lateral malleolus is still a concerning issue [22]. Recent anatomical and biomechanical researches have noted that the augmentation of the IER can provide a similar outcome to that of the calcaneofibular ligament (CFL) in stabilizing the subtalar joint [4, 31]. According to long-term research of arthroscopic-assisted Broström-Gould repair, no obvious limitation of eversion, inversion and degeneration of the subtalar joint was encountered [14]. In the present study, only 3 female cases complained about irritation around the released site of the IER at the early stage due to the keloid formation. Fortunately, after six months postoperative without a second intervention, this problem was noticeably relieved. Also, no recorded stiffness and degeneration of the subtalar joint was encountered in our cases at the final follow-up (Fig. 6D). As mentioned previously, the appropriate tension of the attached IER could avoid tightness of the subtalar joint.

There are several limitations in this study. First, the sample size is relatively small. Second, it remains to be expounded due to the lack of a control group. Further research is needed to compare the difference between endoscopic IER augmentation and open IER augmentation. Third, this is a preliminary report because of a relatively short follow-up time. Clinical trials are needed to further confirm the results in a long-term postoperative period. Additionally, it is difficult to ask every patient to have the stress radiograph in the department of radiology at each follow-up. So, the stress test was performed and evaluated in the department of orthopedics using fluoroscopy. Finally, those special cases with an insufficient ATFL remnant (Type-4) or generalized ligamentous laxity were not included in this present study. This study still provides useful insight into the clinical efficacy of the all-inside arthroscopic Broström-Gould procedure.

## Conclusion

The arthro-Broström procedure combined with endoscopic retinaculum augmentation using all-inside lasso-loop techniques is reliable and safe due to satisfactory clinical results and avoiding nerve injuries. Accurate separation and attachment of the IER under endoscopic visualization could lastingly protect the repaired ATFL remnant without disturbing the subtalar joint.

## Data Availability

The datasets used during the current study are available from the corresponding author on reasonable request.

## Abbreviations

ATFL: Anterior talofibular ligament
CFL: Calcaneofibular ligament
IER: Inferior extensor retinaculum
CAIT: Cumberland Ankle Instability Tool
VAS: Visual Analogue Scale
MRI: Magnetic resonance imaging
US: Ultrasonography
OCL: Osteochondral lesion
LM: Lateral malleolus
AM: Anteromedial
AL: Anterolateral
acAL: Accessory anterolateral
PEEK: Poly-ether-ether-ketone
BMI: Body mass index
CI: Confidence intervals
